# Influence of Excipient Composition on Survival of Vaginal Lactobacilli in Electrospun Nanofibers

**DOI:** 10.3390/pharmaceutics14061155

**Published:** 2022-05-28

**Authors:** Spase Stojanov, Julijana Kristl, Špela Zupančič, Aleš Berlec

**Affiliations:** 1 Department of Biotechnology, Jožef Stefan Institute, Jamova 39, SI-1000 Ljubljana, Slovenia; spase.stojanov@ijs.si; 2 Faculty of Pharmacy, University of Ljubljana, Aškerčeva 7, SI-1000 Ljubljana, Slovenia; julijana.kristl@ffa.uni-lj.si (J.K.); spela.zupancic@ffa.uni-lj.si (Š.Z.)

**Keywords:** vaginal probiotics, electrospinning, nanofibers, viability, lyoprotectants, carbohydrates, delivery system

## Abstract

The lack of appropriate delivery systems hinders the use of probiotics in the treatment of vaginal infections. Therefore, the development of a new delivery system for the local administration of vaginal probiotics is necessary. In this study, we selected three vaginal lactobacilli, i.e., *Lactobacillus crispatus*, *Lactobacillus gasseri*, and *Lactobacillus jensenii*, and incorporated them into nanofibers using electrospinning. Polyethylene oxide (PEO) was used as a carrier polymer to produce nanofibers. It was supplemented with alginate and sucrose selected from a group of carbohydrates for their growth-promoting effect on lactobacilli. The interaction between excipients and lactobacilli was evaluated thermally and spectroscopically. Bacterial survival in polymer solutions and in nanofibers immediately after electrospinning and after storage varied among species and was dependent on the formulation. Sucrose improved the survival in polymer solutions and preserved the viability of *L. crispatus* and *L. jensenii* immediately after electrospinning, and *L. gasseri* and *L. jensenii* during storage. Blending PEO with alginate did not improve species viability. However, the three lactobacilli in the nanofibers retained some viability after 56 days, indicating that composite multifunctional nanofibers can maintain the viability of vaginal lactobacilli and can be used as a potential solid delivery system for vaginal administration of probiotics.

## 1. Introduction

The consumption of probiotics in adequate amounts has been shown to be beneficial to the health and well-being of the host [1]. Probiotics are usually consumed in foods such as yogurt, cheese, ice cream, and cereal or as lyophilized tablets [1]. They are live microorganisms that interact with the microbiota in different niches of the human body. Interest in vaginal probiotics has grown due to their positive effects in the treatment of vaginal infections. In healthy women, the vagina is colonized by the normal microbiota (10^7^–10^8^ CFU/g vaginal fluid), with bacteria from the genus *Lactobacillus* predominating [2]. Vaginal infections, such as bacterial vaginosis, trichomoniasis, and vulvovaginal candidiasis, are caused by dysbiosis in which the number of normally present lactobacilli decreases, resulting in a vaginal pH higher than 4.5, the normal vaginal pH of women in reproductive age. This allows for the overgrowth of opportunistic pathogens [3] and leads to vaginitis, which can result in vaginal discharge, itching, and pain [4]. Although antimicrobials represent the first choice of treatment against vaginal infections, their continued use can lead to resistant pathogens and an approximately 50% infection recurrence rate [3]. The combined therapy of antibiotics (metronidazole) and oral probiotics was more effective than antibiotics alone in treating vaginal infections [5]. Administration of *Lactobacillus* bacteria as probiotics can thus restore the vaginal microbial balance and help cure the existing infections [6,7,8].

Most probiotic formulations are developed for oral administration. Vaginal probiotics can be administered orally and reach the vagina through the rectum [2]; however, this takes longer time and results in a lower number of viable bacteria at the site of action [9]. Therefore, vaginal administration is recommended. However, current dosage forms for vaginal administration have limitations such as: discomfort, short residence time, imprecise dosing, leakage, and variable drug distribution [10]. Additionally, probiotics need to be stable in the formulation and active in the host. Vaginal lactobacilli showed good viability when stored at −70 °C [11], but little is known about their viability in pharmaceutical formulations.

Over the years, various encapsulation techniques for probiotics have been developed to provide protection for bacterial cells [12,13]. Nanofibers produced by electrospinning can serve as a potential drug delivery system [14,15,16] for the nasal, oral, and vaginal mucosa [17] and have several advantages, such as high incorporation efficiency, high surface-to-volume ratio, and good bioavailability. They can provide simultaneous delivery of diverse therapeutics, have good mechanical resistance, and are cost-effective [14]. Nanofibers are soft, non-abrasive, and highly flexible. Although the final form and size of the nanofiber formulation have yet to be determined, it is expected to be administered in the vaginal cavity in the form of a film, capped cylinder, or with a tampon applicator [18]. Incorporating probiotics in nanofibers enables concomitant drying of bacteria and preparation of solid dosage forms in geometries that could directly favor vaginal administration (tampon-like).

In electrospinning, a high voltage is applied to a polymer solution which is pumped through a syringe needle, causing polymer fluid motion, stretching of the polymer, and evaporation of the solvent, resulting in the production of nanofibers [16]. Incorporating probiotic bacteria into nanofibers has gained a lot of interest in recent years [19,20,21,22]. However, only a few studies described the encapsulation of vaginal probiotics [23,24,25]. Three different vaginal *Lactobacillus* species (*L. crispatus*, *L. gasseri*, and *L. jensenii*) were genetically modified to express fluorescent proteins with different spectral properties and incorporated into polyethylene oxide (PEO) nanofibers [23]. Vaginal isolate *Lactobacillus acidophilus* was incorporated into nanofibers using polyvinyl alcohol and polyvinylpyrrolidone with different molecular masses. The viability depended on the storage conditions, with long-term stability observed when storing at or below 7 °C. [24]. Similarly, Silva et al. reported better survival of vaginal *L. gasseri* CRL1320 and *Lactobacillus rhamnosus* CRL1332 incorporated into polyvinyl alcohol when stored at lower temperatures (−20 °C) [25].

Specific excipients added to the nanofibers can act as lyoprotectants or prebiotics. Lyoprotectants prevent cell damage by protecting the protein structure of bacteria based on the “Water Replacement Hypothesis” [26], while prebiotics enhance bacterial growth. Excipients such as sucrose, trehalose, skim milk, lactose, and glycerol have been shown in previous studies to improve the survival of lactobacilli when added to nanofibers [20,25,27]. Polymers used for electrospinning can also affect the survival of probiotics. Natural polymers such as polysaccharides can act as prebiotics and promote the activity and growth of probiotics [28,29]. However, the formation of nanofibers from natural polymers is challenging due to their low stability, different molecular weight, and presence of charged groups. For these reasons, it has been proposed to modify their chemical structures or blend them with synthetic polymers to improve the electrospinning process [30].

In the present study, the aim was to develop nanofibers into which three live strains of vaginal lactobacilli (*L. gasseri*, *L. crispatus*, and *L. jensenii*) commonly found in the vagina were separately incorporated. The initial focus was to select the most promising excipients for the nanofiber formulations based on the growth characteristics (lag time, growth rate, and maximum optical density (OD)) of the lactobacilli in the presence of various polymers and lyoprotectants. This led to the addition of sodium alginate (hereafter referred to as alginate) and sucrose, which were the most effective prebiotics in vitro, to the main carrier polymer PEO. The survival of the lactobacilli was evaluated in liquid polymer suspensions and in dry nanofiber mats, whereby the latter were also analyzed physico-chemically to assess excipients’ crystallinity and interactions with bacteria. The identity of the species after their release from the nanofibers was confirmed by colony PCR.

## 2. Materials and Methods

### 2.1. Bacterial Storage and Culturing

Three vaginal species from the genus *Lactobacillus* were used in this study: *L. crispatus* ATCC 33820, *L. gasseri* ATCC 33323, and *L. jensenii* ATCC 25258. They were kept frozen at −80 °C in De Man, Rogosa, and Sharpe medium (MRS; Merck, Darmstadt, Germany), supplemented with 20% glycerol for long-term storage. Bacteria were transferred from the frozen stocks to solidified MRS media containing 1.5% agar and grown at 37 °C for 2–3 days in anaerobic bags (GasPak^TM^ EZ; Becton Dickinson, Franklin Lakes, NJ, USA) or jars (AnaeroGenTM 2.5l; Thermo Scientific, Waltham, MA, USA). Three to four colonies were transferred to 10 mL MRS and incubated at 37 °C for one day. These fresh bacterial cultures were inoculated (1:50) in different volumes of MRS supplemented as specified below and incubated at 37 °C for ~16 h.

### 2.2. Growth Characteristics of Lactobacilli in the Presence of Different Excipients

To test the influence of excipients on bacterial growth, MRS was supplemented with different *w*/*v* concentrations of alginate (0.25%, 0.5%, and 1%; Protanal^®^ LF 10/60, Dupont, Copenhagen, Denmark), chitosan (0.1% and 0.2%; Sigma Aldrich, Darmstadt, Germany), α-lactose monohydrate (1%, 2%, and 4%; Sigma Aldrich, Darmstadt, Germany), trehalose dihydrate (1%, 2%, and 4%; Calbiochem, Darmstadt, Germany) and lactulose (1%, 2%, and 4%; Carl Roth, Karlsruhe, Germany). The selected growth media were prepared aseptically using cellulose acetate sterile filters with a pore size of 0.2 µm (Minisart filters; Sigma Aldrich, Darmstadt, Germany). Fresh overnight cultures of *L. gasseri*, *L. crispatus*, and *L. jensenii* were diluted (1:100) in 200 µL MRS media with or without specific polymer/disaccharide. Each strain was incubated in quadruplicate in 96 well microplates sealed with sealing film and grown at 37 °C for 50 h with absorbance measurements every 2 min in a microplate reader (Sunrise; Tecan, Salzburg, Austria) as previously described [19,31]. Growth characteristics (lag time, growth rate, and maximum OD) were analyzed according to the model of Baranyi and Roberts using DMFit 3.5 software [32].

### 2.3. Preparation of Lactobacilli Dispersion in Polymer Solutions

Due to different growth kinetics and different cell concentration in the stationary phase, the three species were grown in different volumes (*L. gasseri* in 400 mL, *L. crispatus* in 800 mL, and *L. jensenii* in 200 mL) to yield a comparable number of cells for viability studies and electrospinning. Dispersions of lactobacilli in polymer solutions were prepared as previously described [19,20,23] with some modifications as follows. The species were grown until reaching their maximal concentration in the stationary phase and were centrifuged at 4900× *g* for 10 min at 4 °C (Rotanta 460R, Hettich, Tuttlingen, Germany). After that, the bacteria were washed once and resuspended in 5 mL water. The 5 mL bacterial suspension (10^10^–10^14^ CFU/mL) was mixed with 5 mL 2-fold-concentrated polymer solutions for 15–30 min at 4 °C to obtain 10 mL homogenous bacterial-polymer suspension with polymer concentrations specified below. Three different polymer solutions were used for the three *Lactobacillus* species, yielding a total of nine combinations. The species were dispersed in final concentrations of 4% (*w*/*v*) PEO (Mw 900 kDa; Sigma Aldrich, Darmstadt, Germany), 4% (*w*/*v*) PEO/alginate (ratio 80/20) and 8% (*w*/*v*) PEO/alginate/sucrose (ratio 40/10/50). Surfactant Tween 80 (1% (*v*/*v*)) was added to PEO/alginate and PEO/alginate/sucrose suspension.

### 2.4. Preparation of Nanofibers

Polymer solutions with and without bacteria were loaded in 5 mL syringes and fixed horizontally to a pump connected to an electrospinning machine (Spinbox, BioInicia SL, Valencia, Spain). The electrospinning conditions for nanofiber production were a flow rate of 150–250 µL/h and an applied voltage of 10–13 kV. Nanofibers were collected on a grounded metal collector covered with aluminum foil 15 cm away from the needle. The temperature and humidity were controlled at ~20 °C and ~30%, respectively.

### 2.5. Scanning Electron Microscopy

The morphology of the nanofibers was observed under a scanning electron microscope (SEM) Supra 35 VP (Carl Zeiss, Oberkochen, Germany). Nanofiber mats with and without bacteria were collected on aluminum foil and attached to the double-sided conductive tape, which was placed onto a metal stub. Imaging of the samples was conducted at a low accelerating voltage (1 kV) with a secondary electron detector. The diameters of 30 randomly selected nanofibers were measured in areas without bacteria and averaged using the ImageJ 1.44p software (National Institutes of Health, Bethesda, MD, USA).

### 2.6. Thermal Analyses

Pure PEO, alginate, and sucrose powders, their physical mixtures in the ratios explained above, nanofibers without bacteria, bacteria lyophilized from water (obtained with Chris Beta 1–8 K; Martin Chris, Osterode am Harz, Germany as previously reported [20]), and nanofibers loaded with bacteria were analyzed by differential scanning calorimetry (DSC; Mettler Toledo, Greifensee, Switzerland) and thermogravimetric analysis (TGA; Mettler Toledo, Greifensee, Switzerland) for their crystallinity and moisture content.

The performance of DSC was assessed with ~5 mg samples that were weighted in aluminum pans with a pinhole. The samples were analyzed between 0 °C and 220 °C, with a heating rate of 10 °C/min and nitrogen flow of 50 mL/min. The results of the obtained curves were normalized according to the sample mass.

Similarly, TGA measurements were performed to evaluate the moisture content of the samples. Approximately 5 mg of the sample mass was analyzed from 30 °C to 220 °C, with a heating rate of 10 °C/min and nitrogen flow of 50 mL/min.

### 2.7. Fourier Transform Infrared Spectroscopy (FTIR)

Fourier transform infrared spectroscopy (FTIR) was utilized to evaluate the interaction between bacteria and nanofibers, as well as the interaction between the substances. Like the thermal analyses, pure powder substances, their mixtures, nanofiber mats with and without bacteria, and lyophilized bacteria were used for FTIR spectroscopy at a resolution of 2 cm^−1^ with 64 scans from 4000 cm^−1^ to 600 cm^−1^.

### 2.8. Bacterial Viability in Polymer Solutions

The homogenous polymer solution with bacteria was loaded in syringes and kept at room temperature (24 ± 2 °C) and at 4 °C to mimic electrospinning conditions. Bacterial viability was tested immediately after preparation and after 2, 4, and 6 h of storage, using the drop-plate method [33]. Serial ten-fold dilutions of bacterial-polymer suspensions were prepared in PBS buffer (*L. gasseri*), 0.9% (*w*/*v*) NaCl (*L. crispatus*) or 4% (*w*/*v*) sucrose (*L. jensenii*). Five 10 µL drops of each dilution were pipetted on two MRS agar plates and incubated in anaerobic bags or jars at 37 °C for 2–3 days. Viability was normalized to CFU/g dry mass to enable comparison with nanofibers. For this purpose, the dry mass of the suspension was determined separately by heating the suspension to 100 °C until the water evaporated. The dry mass of the polymer and bacteria and the amount of water were calculated.

### 2.9. Bacterial Viability in Nanofibers after Electrospinning and Long-Term Storage

Suspensions of the three bacterial species in polymers were electrospun to produce nanofiber mats with a mass of 10 ± 2 mg per sample. These were stored at 4 °C and 14% relative humidity. The viability of bacteria in the nanofiber mats was assessed immediately after preparation and 7, 14, 21, 28, and 56 days after by dissolving the nanofiber in PBS buffer (*L. gasseri*), 0.9% (*w*/*v*) NaCl (*L. crispatus*) or 4% (*w*/*v*) sucrose (*L. jensenii*), and performing the drop plate assay, as described in Section 2.8. Viability was normalized to the mass of nanofibers (CFU/g).

### 2.10. Colony PCR for Bacterial Identification after Nanofiber Dissolution

Colony PCR was performed to confirm the identity of bacterial colonies grown on solidified MRS media after their release from nanofibers. Primers (Integrated DNA Technologies) were designed to amplify a short genomic DNA fragment (200–400 base pairs) identified using IMG/M (https://img.jgi.doe.gov/m/ (accessed on 23 May 2022)) and were specific for each of the species tested (Table 1). Bacterial colonies were transferred to a PCR mixture (DreamTag^TM^ DNA Polymerase, Thermo Scientific, Waltham, MA, USA). The samples were heated at 99 °C for 10 min to lyse the bacteria, followed by the addition of Tag DNA Polymerase (Thermo Scientific). The PCR conditions were as follows: denaturation at 94 °C for 30 s, annealing at 56 °C for 1 min, and extension at 72 °C for 1 min for 30 cycles, followed by a 5 min extension at 72 °C. After the PCR reaction, the samples were loaded in 1.5% agarose (Sigma Aldrich, Darmstadt, Germany) gel and visualized under UV light. Positive control from frozen stocks and negative control without bacteria were also included.

### 2.11. Statistical Analysis

The statistical significance was analyzed with Prism 5.00 (GraphPad software, San Diego, CA, USA), using Student’s *t* test and a one-way ANOVA with Bonferroni correction. The results were presented as means ± standard deviation (SD).

## 3. Results

### 3.1. Primary Screening of the Effect of Carbohydrates on the Growth of Vaginal Lactobacilli

**Figure 1:**
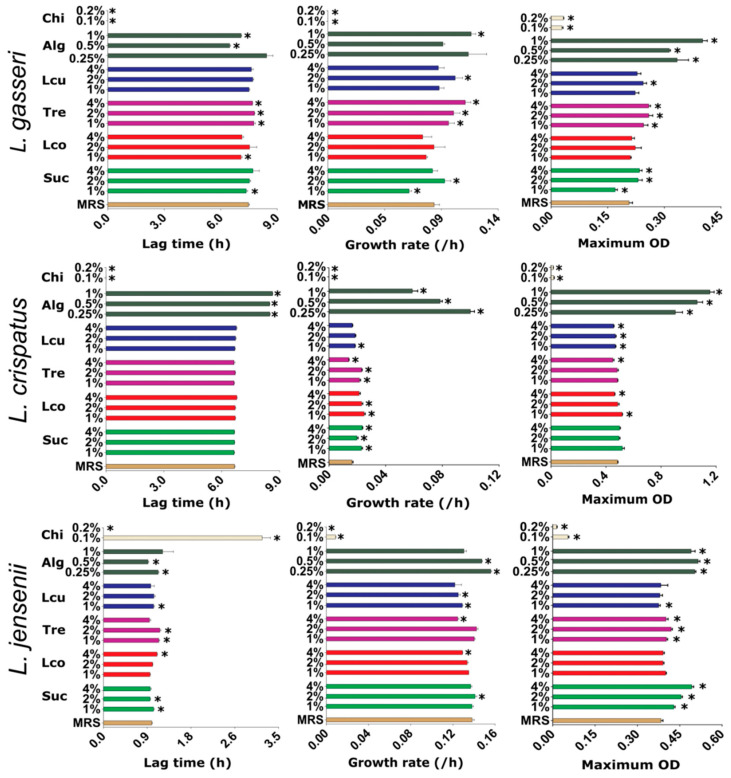
Growth characteristics (lag time, growth rate, and maximum optical density (OD)) of vaginal lactobacilli *L. gasseri*, *L. crispatus*, and *L. jensenii* cultured under the same conditions in sterile filtered MRS with the addition of one carbohydrate with different concentrations (*w*/*v*): sucrose (Suc), lactose (Lco), trehalose (Tre), lactulose (Lcu), alginate (Alg), or chitosan (Chi). *, *p* < 0.05 (Student’s *t* tests, relative to MRS control).

To identify the most promising excipient acting as a prebiotic in all three lactobacilli species, their growth characteristics (lag time, growth rate, and maximum OD) were determined in the presence of different concentrations of polymers (alginate and chitosan) and disaccharides (sucrose, lactose, trehalose, and lactulose) (Figure 1). The excipients mostly had different effects on the growth of the three species when cultivated under the same conditions. However, some common influences of the excipients on bacterial growth were observed. For example, the two polymers, both polysaccharides, had the most pronounced but opposite effect on the growth characteristics of all lactobacilli species, with alginate promoting and chitosan strongly inhibiting their growth. However, while alginate increased the maximum OD by 1.9-fold and the growth rate by 1.3-fold, it also delayed the onset of bacterial growth, particularly in *L. crispatus*, resulting in an average increase of 1.3 h in lag time. The inhibition of *Lactobacillus* growth by chitosan has been observed previously [34] and completely prevents its potential use as an excipient for nanofiber production with lactobacilli. Sucrose also stimulated growth, with an increase in the maximum OD observed in *L. gasseri* and *L. jensenii*. In *L. jensenii*, the addition of 4% sucrose resulted in a 1.2-fold increase, and *L. gasseri* in a 1.1-fold increase in maximum OD. Trehalose slightly stimulated the growth of *L. gasseri* and *L. jensenii* but showed no effect on *L. crispatus*. Trehalose also prolonged the lag time of *L. gasseri* and *L. jensenii* while increasing the growth rate of *L. gasseri* and *L. crispatus*. Both lactose and lactulose did not stimulate or inhibit bacterial growth and showed little effect on lag time and growth rate. Alginate and sucrose demonstrated the best prebiotic characteristics in vitro and were therefore used in the nanofiber formulations.

### 3.2. Bacterial Viability in Different Polymer Solutions

**Figure 2:**
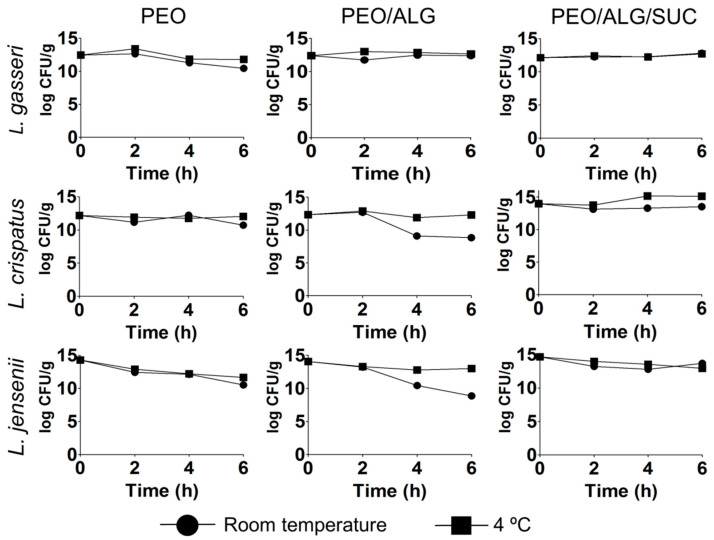
Viability (log CFU/g) of *L. gasseri*, *L. crispatus*, and *L. jensenii* in polyethylene oxide (PEO), polyethylene oxide/alginate (PEO/ALG), and polyethylene oxide/alginate/sucrose (PEO/ALG/SUC) at different time points and at different temperature.

Bacterial viability in different polymer solutions, kept at 4 °C and room temperature, was studied at different time points (0–6 h) to evaluate the possible decrease in viability during the electrospinning process, which can take several hours. All species demonstrated better survival when sucrose was added and incubated at 4 °C (Figure 2). When incubated at room temperature, the viability of *L. gasseri* remained stable in PEO/alginate solution, while the viability in PEO decreased by 2 log CFU/g after 6 h. For *L. crispatus*, the situation was reversed; the viability of the strain was more stable in PEO solution, while in PEO/alginate, it decreased by 3.5 log CFU/g, whereas in PEO/alginate/sucrose, it decreased by 0.4 log CFU/g. After 6 h of incubation, the worst survival was observed for *L. jensenii* at room temperature, with a decrease in viability of 3.7 log CFU/g when dispersed in PEO and of 5.2 log CFU/g when dispersed in PEO/alginate. Here, sucrose improved the viability considerably, with a decrease only of ~1 log CFU/g after 6 h of incubation at both room temperature and 4 °C. To sum up, the lactobacilli viability in polymer solution can decrease up to 5.2 log CFU/g at room temperature, but this can be prevented by the addition of 50 % (*m*/*m*) sucrose to the polymer solution.

### 3.3. Nanofiber Morphology

**Figure 3:**
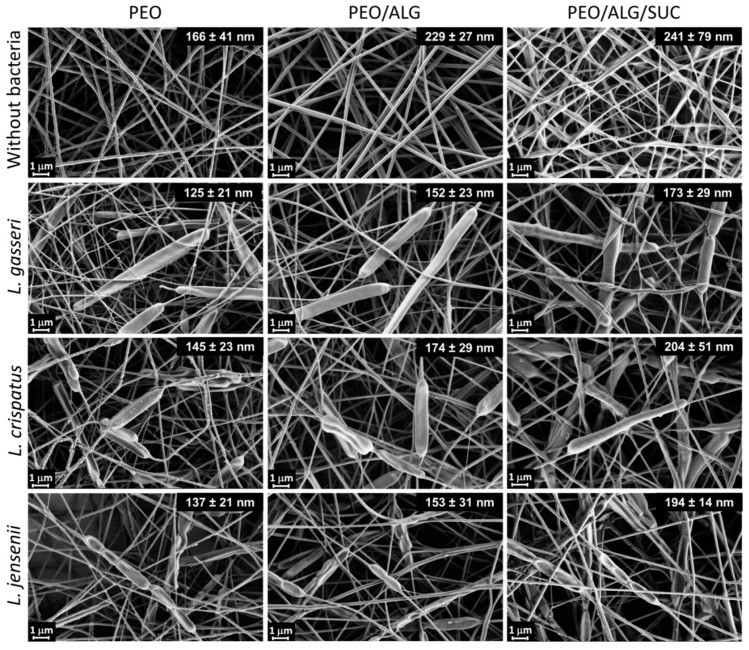
Scanning electron microscopy images of polyethylene oxide (PEO), polyethylene oxide/alginate (PEO/ALG), and polyethylene oxide/alginate/sucrose (PEO/ALG/SUC) nanofibers without bacteria, or containing *L. gasseri*, *L. crispatus*, and *L. jensenii*. Average diameters of nanofibers are depicted in upper right corners of the images.

Nanofibers with and without bacteria were successfully developed, as can be observed from SEM images (Figure 3). The mean diameter of PEO nanofibers without bacteria was 166 ± 41 nm, while the addition of alginate and alginate/sucrose to PEO significantly increased diameter to 229 ± 27 nm and 241 ± 79 nm, respectively. The incorporation of lactobacilli cells was observed with specific thickenings of the nanofibers, as shown previously [23]. Lactobacilli decreased the mean diameter of nanofibers, which is contrary to our previous findings [23]. This may be due to the use of a different electrospinning machine (Fluidnatek LE100; BioInicia SL, Valencia, Spain) or higher conductivity of the sample due to higher ion concentration retained after the lactobacilli preparation procedure. The diameter of the nanofibers also differed within the samples, especially when sucrose was added. The addition of sucrose caused the fusion of nanofibers during drying, which is more evident in the absence of bacteria.

### 3.4. Crystallinity and Moisture Content in Nanofibers

**Figure 4:**
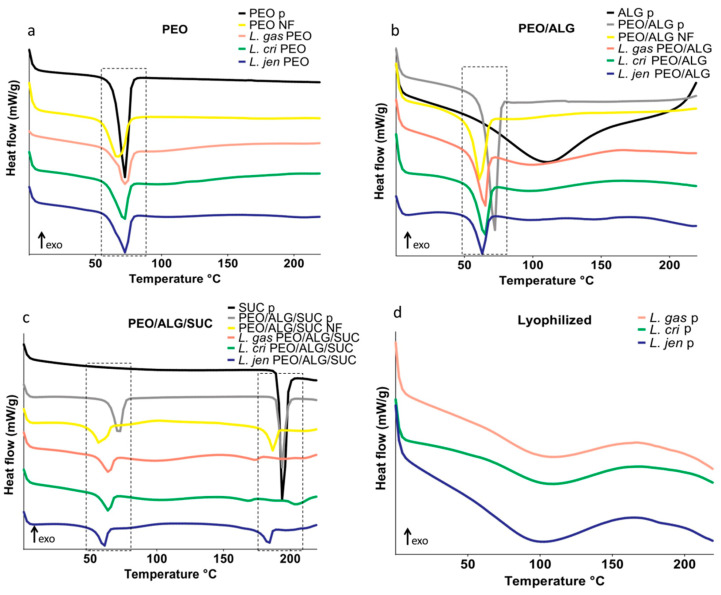
DSC thermograms of polyethylene oxide (PEO) (**a**), PEO/alginate in 80/20 ratio (PEO/ALG) (**b**), and PEO/alginate/sucrose in 40/10/50 ratio (PEO/ALG/SUC) (**c**) in powder form (p), and in nanofibers (NF) without or with *L. gasseri* (*L. gas*), *L. crispatus* (*L. cri*), and *L. jensenii* (*L. jen*). (**d**) DSC thermograms of lyophilized lactobacilli.

The crystallinity of pure PEO, alginate, sucrose, their physical mixtures, and nanofibers with and without bacteria was analyzed thermally by DSC and TGA (Figure 4 and Table 2). The melting temperature of pure PEO powder was 70.8 °C. PEO nanofibers melted at a slightly lower temperature (66.4 °C) with lower melting enthalpy than the PEO powder, indicating decreased crystallinity after electrospinning. PEO in PEO nanofibers with lactobacilli was also less crystalline than the PEO powder (Figure 4a). Pure alginate powder is a semi-crystalline polymer with a broad endothermic peak indicating water evaporation, which is also confirmed by TGA. Alginate in PEO/alginate nanofibers with and without lactobacilli contributed to an additional decrease in PEO melting temperature (60.4 °C) and crystallinity compared to their physical mixture (Figure 4b). Sucrose powder melted at 191.9 °C. In the physical mixture of PEO/alginate/sucrose, two endothermic peaks were visible when PEO and sucrose melted at 70.5 °C and 193.1 °C, respectively. Electrospinning reduced the crystallinity of sucrose in PEO/alginate/sucrose nanofibers, and sucrose was almost completely amorphized when lactobacilli, especially *L. gasseri* and *L. crispatus*, were incorporated into the nanofibers (Figure 4c). Lyophilized lactobacilli in the powder showed broad peaks at about 110 °C, indicating the evaporation of water (Figure 4d).

Moisture content differed between samples. In the PEO and sucrose powders, the moisture content was 0%, while in alginate it was 12.3%. In nanofibers with alginate, the moisture content was higher than that in PEO nanofibers. All three lyophilized species had a similar moisture content of about 5%, whereas nanofibers with lactobacilli had a moisture content of 1.7–4.1%, more than nanofibers without bacteria (Table 2).

### 3.5. Interaction between Lactobacilli and Excipients

**Figure 5:**
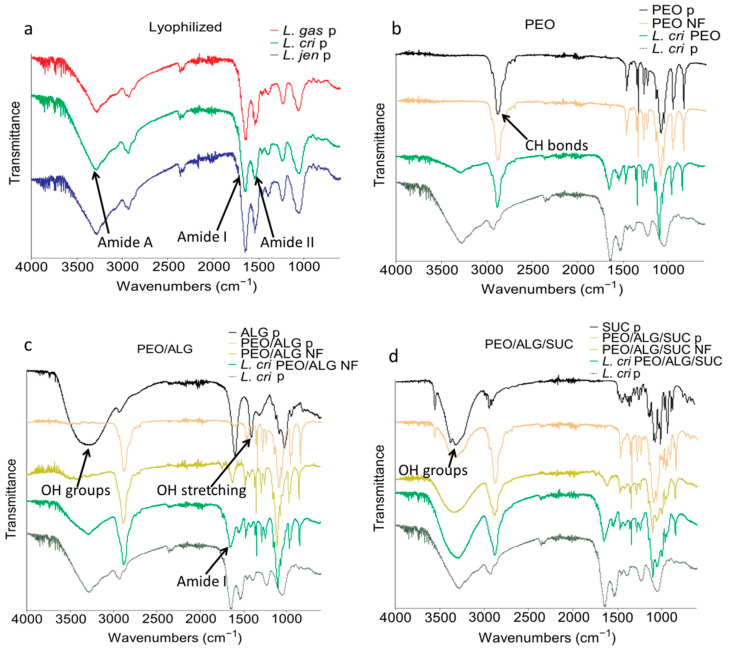
Fourier transform infrared (FTIR) spectra of lyophilized lactobacilli (**a**), polyethylene oxide (PEO) (**b**), PEO/alginate in 80/20 ratio (PEO/ALG) (**c**), and PEO/alginate/sucrose in 40/10/50 ratio (PEO/ALG/SUC) (**d**) in powder form (p), and in nanofibers (NF) without or with *L. gasseri* (*L. gas*), *L. crispatus* (*L. cri*), and *L. jensenii* (*L. jen*).

The interaction between excipients and bacteria was assessed using FTIR spectroscopy (Figure 5). Lactobacilli that were either lyophilized or incorporated into nanofibers demonstrated three amide bands (Amide I, Amide II, and Amide A), characteristic of proteins in bacteria. The three lyophilized species demonstrated similar peaks (Figure 5a) at 1530 cm^−1^ (Amide II), 1640 cm^−1^ (Amide I), and 3270 cm^−1^ (Amide A). Amide I region represents the C=O stretching of the peptide bonds, which is related to the secondary structure of the proteins. At the same time, Amide II and Amide A the N-H bending and N-H stretching, respectively [35,36]. Specific amide bands representing vibration in amide bonds from bacterial proteins were not detected in the powder or the nanofiber mats without bacteria. PEO showed two peaks at 840 cm^−1^ and 960 cm^−1^, representing C-H bending. A large peak at around 1090 cm^−1^ was also observed, confirming the C-O-C stretching, while two small peaks at 1240 cm^−1^ and 1280 cm^−1^ confirmed symmetric C-H_2_ twisting. Peaks at 1340 cm^−1^ and 1470 cm^−1^ correspond to asymmetric C-H_2_ bending and C-H_2_ scissoring. An additional narrow peak at around 2900 cm^−1^ was also observed, confirming the C-H bonds in the alkane chain. Because of the similarity of the graphs of the lyophilized bacteria, only *L. crispatus* incorporated into different nanofiber mats is shown in Figure 5. The incorporation of *L. crispatus* resulted in additional peaks at 1540 cm^−1^, 1650 cm^−1^, and 3270 cm^−1^, confirming the Amide II, Amide I, and Amide A regions, respectively. Incorporated bacteria have shifted the peak at 1090 cm^−1^ to 1100 cm^−1^, 1530 cm^−1^ to 1540 cm^−1^, and 1640 cm^−1^ to 1650 cm^−1^, suggesting an interaction in hydrogen bonding between bacteria and PEO (Figure 5b).

Pure alginate powder showed a small peak at 940 cm^−1^ and a larger peak at 1024 cm^−1^, corresponding to C-O stretching. Other peaks were detected at 1407 cm^−1^ and 1590 cm^−1^ due to the stretching vibration of COO- salts. A small peak at 2900 cm^−1^ and broad peak at 3240 cm^−1^, indicating the presence of OH groups, were also observed in pure alginate powder but not when alginate was mixed with PEO in powder form and the nanofiber formulation. Mixing PEO with alginate resulted in the shifting of the 1590 cm^−1^ peak to 1610 cm^−1^, while the incorporation of *L. crispatus* resulted in the reappearance of Amide I and Amide II peaks and their shift to 1640 cm^−1^ and 1540 cm^−1^ respectively. A shift also occurred in the Amide A region, with the peak at 3270 cm^−1^ moving to 3280 cm^−1^, suggesting an interaction in O-H stretching within the carboxylic acid. Like PEO, shifting in the peak at 1090 cm^−1^ to 1100 cm^−1^ was also observed (Figure 5c).

Sucrose alone and its physical mixture with PEO and alginate showed small peaks at 630 cm^−1^, 670 cm^−1^, and 730 cm^−1^, but these were eliminated in nanofiber mats. Similarly, two separate peaks at 3320 cm^−1^ and 3380 cm^−1^ were observed in pure sucrose and its physical mixture with PEO and alginate. However, they merged into a broad peak at 3330 cm^−1^ when sucrose was incorporated into nanofibers. Incorporating bacteria into the nanofibers resulted in in the Amide I, Amide II, and Amide A regions shifting, namely from 1630 cm^−1^ to 1640 cm^−1^, 1530 cm^−1^ to 1540 cm^−1^, and at 3270 cm^−1^ to 3280 cm^−1^, respectively. As with the other formulations, the incorporation of bacteria shifted the peak from 1090 cm^−1^ to 1100 cm^−1^ (Figure 5d).

### 3.6. Viability of Vaginal Lactobacilli in Different Nanofiber Formulations Immediately after Incorporation and after Long-Term Storage

**Figure 6:**
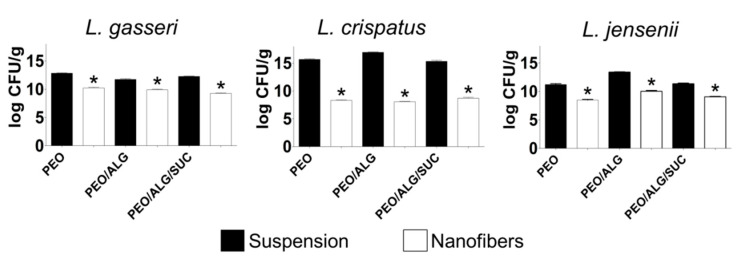
Impact of electrospinning on the viability of vaginal lactobacilli *L. gasseri*, *L. crispatus*, and *L. jensenii*. Black bars denote viability in polymer suspension normalized to the dry mass of components in suspension. White bars denote viability in nanofibers. PEO, polyethylene oxide; ALG, alginate; SUC, sucrose. * *p* < 0.05 (Student’s t tests) relative to bacterial suspension.

The electrospinning of the three vaginal lactobacilli, *L. gasseri*, *L. crispatus*, and *L. jensenii*, resulted in a significant decrease in viability immediately after electrospinning compared with a bacterial suspension (Figure 6). The sensitivity of lactobacilli to the stresses encountered during electrospinning is species-specific, with *L. crispatus* being the most sensitive and *L. gasseri* the least sensitive. Sucrose was able to protect *L. crispatus* and *L. jensenii* during electrospinning. However, it showed negative effects on *L. gasseri* (3.0 log CFU/g decrease in PEO/alginate/sucrose, compared to 1.8 log CFU/g decrease in PEO/alginate). The viability of *L. crispatus* decreased strongly (by 8.8 log CFU/g) after electrospinning in PEO/alginate but was improved by the addition of sucrose (decrease by 6.6 log CFU/g). Electrospinning of *L. jensenii* in PEO/alginate/sucrose resulted in a 2.2 log CFU/g decrease, while in PEO/alginate, the viability decreased by 3.3 log CFU/g. Nevertheless, vaginal lactobacilli were able to survive the electrospinning, with *L. gasseri* demonstrating the highest survival, followed by *L. jensenii* and *L. crispatus*.

**Figure 7:**
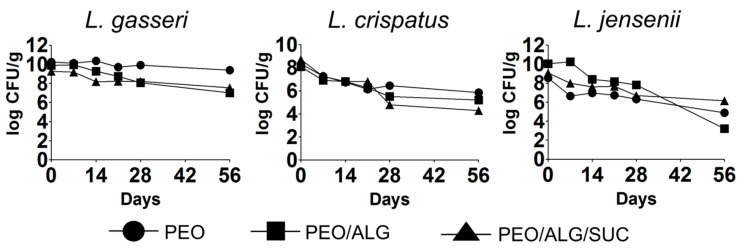
Long-term viability of vaginal lactobacilli *L. gasseri*, *L. crispatus*, and *L. jensenii* incorporated into different nanofiber formulations and stored at 4 °C and 14% relative humidity. PEO, polyethylene oxide; ALG, alginate; SUC, sucrose.

The long-term viability of the vaginal lactobacilli after their incorporation into nanofibers is crucial for their optimal therapeutic effect. The three species retained viability in nanofibers for 56 days when stored at 4 °C, albeit a considerable drop in viability was observed with *L. crispatus* and *L. jensenii* (Figure 7). *L. gasseri* in PEO nanofibers was the most stable, with a decrease in viability of 0.8 log CFU/g after 56 days. Mixing alginate with PEO in the nanofibers reduced the viability by 2.8 log CFU/g, while the addition of sucrose in PEO/alginate resulted in a 1.7 log CFU/g viability decrease. The incorporation of *L. jensenii* into PEO nanofibers resulted in greater viability after 56 days (3.6 log CFU/g decrease) in comparison to PEO/alginate nanofibers, where the viability decreased by 6.8 log CFU/g. Like *L. gasseri*, the addition of sucrose to PEO/alginate resulted in greater stability of incorporated *L. jensenii*, with a 2.9 log CFU/g viability decrease after 56 days. Interestingly, after 28 days, the viability of *L. jensenii* was greater in PEO/alginate in comparison to the other two formulations. The incorporation of *L. crispatus* into pure PEO nanofibers resulted in the highest viability (2.4 log CFU/g decrease after 56 days). Addition of alginate impaired the viability of *L. crispatus* (decrease by 2.8 log CFU/g in PEO/alginate), which was further impaired by the addition of sucrose (decrease in viability by 4.4 log CFU/g in PEO/alginate/sucrose after 56 days).

### 3.7. Identification of Vaginal Lactobacilli after Release from Nanofibers

**Figure 8:**
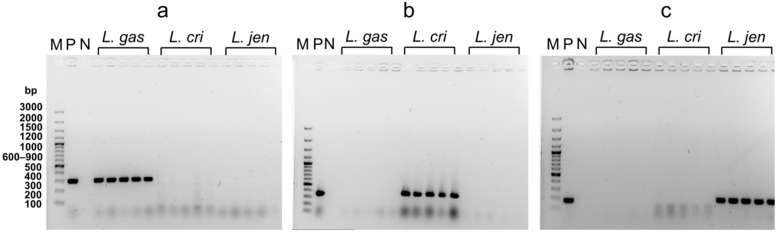
Agarose gel electrophoresis of colony PCR, using specific primers for *L. gasseri* (**a**), *L. crispatus* (**b**), and *L. jensenii* (**c**). DNA size marker (M), positive control (P), negative control (N), *L. gasseri* (*L. gas*), *L. crispatus* (*L. cri*), and *L. jensenii* (*L. jen*).

After their release from nanofibers, the identity of lactobacilli was confirmed with colony PCR that produced species-specific amplicons of the correct size. A representative assay of five colonies of each of the species is depicted in Figure 8. The primers for *L. gasseri* (a) amplified part of the chaperonin (cpn60) gene, for *L. crispatus* (b) part of the gene encoding the GTP-binding protein (lepA), and for *L. jensenii* (c) part of the gene encoding the transketolase subunit A. Positive control from the frozen stocks and negative control without bacteria were also included. The primers showed no cross-reactivity among different lactobacilli species. The sizes of bands in the gel corresponded to the correct sizes of the amplified genes and were the same as that of the positive control, confirming the identity of the bacteria after their release from nanofibers and excluding contamination during the electrospinning procedure.

## 4. Discussion

In this study, we selected three different lactobacilli (*L. crispatus* ATCC 33820, *L. gasseri* ATCC 33323, and *L. jensenii* ATCC 25258) that are dominant in the healthy vagina and are crucial members of the normal vaginal microbiota. Vaginal lactobacilli, compared to other lactic acid bacteria, are characterized by low viability and high sensitivity to environmental factors, mainly tonicity and oxygen. We, therefore, tested their growth characteristics in the presence of different natural polymers and disaccharides. Lactobacilli can metabolize disaccharides by different pathways and thus use different carbon sources to promote their growth [37]. In a recent study, we showed that the carbohydrate-rich water extract of silver fir could stimulate the growth of some lactobacilli [31]. Disaccharides are also known lyoprotectants and can preserve lactobacilli during drying [38,39]. Here, we observed that sucrose improved the growth of the three lactobacilli, especially *L. jensenii*. Lactulose was shown to promote bacterial growth of vaginal lactobacilli [40]. However, in our study, it showed no prebiotic potential. Polymers had the opposite effect on the growth, with alginate considerably improving and chitosan inhibiting the growth of the three species. The prebiotic potential of alginate may be related to its enzymatic hydrolysis by the bacteria and the production of alginate oligosaccharides which are known prebiotics for lactobacilli [41]. On the other hand, the positively charged groups of chitosan interact with the negatively charged groups of the bacterial membrane and disrupt its membrane permeability [34]. Based on these results, alginate and sucrose were included in electrospinning formulations with PEO as the main carrier polymer. This choice was due to the previous effective incorporation of lactic acid bacteria into PEO nanofibers [19,20,23,42], its biocompatibility and mucoadhesivity, and its inertness with the delivered substances [43]. The multivalent cations and chemical structure of pure alginate limit its use in electrospinning. To overcome this, blending with synthetic polymer is required [44,45,46]. In our preliminary research (not shown), we tested different concentrations of alginate and PEO with different molecular weights. A higher concentration of alginate resulted in droplet formation and non-uniform nanofibers, especially when the polymer was mixed with bacteria. The addition of the surfactant Tween 80 improved the formation of nanofibers due to the decrease of the surface tension of the alginate-containing solution, resulting in the formation of smoother nanofibers [47].

Lactobacilli are microaerophilic or anaerobic bacteria, and their exposure to oxygen is damaging to the cells [48,49]. Similarly, the hypotonic environment [50] negatively affects their viability which prompted us to test different solutions in the washing process before final resuspension in water. *L. gasseri* was more viable when washed with PBS, *L. crispatus* with 0.9% (*w*/*v*) NaCl, and *L. jensenii* with 4% (*w*/*v*) sucrose solution (data not shown). However, ionic compounds interfere with the electrospinning procedure and cause instabilities in the electrospinning jet due to high electrical conductivity [51]. For this reason, the final suspension of bacteria for electrospinning was in pure water to obtain uniform nanofibers without interfering with the ions. The effects of oxygen and hypotonic environments on lactobacilli viability were evaluated at different time points and different temperatures. In a hypotonic environment, bacteria absorb water leading to their swelling and lysis. Sucrose increases the osmolarity of the solution and prevents water from crossing the bacterial membrane. The lactobacilli showed better survival with the addition of sucrose and when incubated at 4 °C. Lower temperatures are also critical for the survival of vaginal lactobacilli during electrospinning. For these reasons, we washed the bacteria only once and used lower temperatures during electrospinning. Reducing the number of washing steps results in higher ion concentrations in the final suspension, leading to higher conductivity and thinner nanofibers [52]. In the current study, the incorporation of bacteria into the nanofibers resulted in a smaller mean diameter which is contrary to the previous findings, where we observed thicker nanofibers when lactobacilli were incorporated [23]. In our previous research, we performed two washing steps with pure water to completely remove the ions and incorporated the engineered vaginal lactobacilli at a higher temperature (37 °C) with vertical electrospinning. The different procedures and machines affected the mean diameter of nanofibers when lactobacilli were incorporated. Despite aseptic working conditions, contamination may occur [53]. The identity of bacteria after their release from nanofibers can be confirmed by PCR which is a method that can detect the presence or absence of specific microorganisms with high sensitivity [54]. The identity of the bacteria after nanofiber dissolution was confirmed with colony PCR [55] using species-specific primers.

Electrospinning of the polymers resulted in lower enthalpy and reduced crystallinity in nanofibers. In its powder form, sucrose demonstrated the highest crystallinity, which was also reduced with electrospinning, especially with the addition of lactobacilli. Amorphization of sucrose can stabilize the cell membrane of the bacteria and result in better viability after long-term storage, which was observed with *L. gasseri* and *L. jensenii*. Electrospinning of polymers also resulted in lower melting temperatures and moisture content. This may be linked to the interaction between the bacteria and excipients in nanofibers, as was previously suggested [20]. The moisture content of the three lyophilized species was lower compared to lyophilized *L. plantarum* [20]. Nevertheless, the moisture content of lactobacilli decreased when encapsulated in nanofibers. Interaction between lactobacilli and excipients and excipients with each other was also observed with FTIR, which is a sensitive method for investigating probiotics and their interactions [56]. The three lyophilized species showed similar spectra, indicating that similar functional groups are present in the bacteria. The similarity of the spectra cannot explain the difference in bacterial viability among the species. The incorporation of vaginal lactobacilli into nanofibers resulted in a shift of several peaks in the spectra. The peak shift from 1090 cm^−^^1^ to 1100 cm^−^^1^ could be due to the hydrogen bonds between the polyether group of PEO and amino or hydroxyl groups of bacteria [57]. Interaction between the bacterial proteins in the Amide I and Amide II region with PEO was observed by a shift from 1530 cm^−^^1^ to 1540 cm^−^^1^ and from 1640 cm^−^^1^ to 1650 cm^−^^1^, respectively. Interaction in the Amide A region was observed only with alginate and sucrose, with a shift in the peak at 3270 cm^−^^1^ to 3280 cm^−^^1^. The OH groups in sucrose and alginate interact with the membrane proteins of bacteria. The addition of sucrose to the nanofibers loaded with bacteria resulted in hydrogen bonding with the membrane proteins and protecting the bacteria during electrospinning and long-term storage [26]. This explains the protective effect of sucrose on some species. Viability was tested in polymer solutions immediately after electrospinning and in nanofibers during storage. Viability in polymer solutions may be associated with lower tolerance to hypotonic environment and oxygen, with *L. jensenii* being the most sensitive. The addition of sucrose preserved the viability and improved the survival of the three species in polymer solution by increasing the osmolarity of the environment. In addition, sucrose acted as a lyoprotectant by preserving the viability of *L. crispatus* and *L. jensenii* during electrospinning. All the species retained viability after 56 days of storage in nanofiber mats. However, the viability of encapsulated bacteria in nanofibers during long-term storage also differed between species. *L. gasseri* demonstrated the highest survival rate, and *L. crispatus* the lowest. Addition of sucrose improved viability and resulted in better survival of *L. gasseri* and *L. jensenii* but impaired the survival of *L. crispatus*. The addition of alginate in the nanofiber formulations did not contribute to bacterial preservation, especially after longer periods, where pure PEO was shown to be more effective. Different lactobacilli survived differently in the same formulations; therefore, incorporation of vaginal lactobacilli individually rather than in a mixture is preferable. A tailored formulation is required for each species to obtain a sustainable number of viable bacteria.

## 5. Conclusions

The susceptibility of vaginal lactobacilli to environmental factors and the lack of a suitable delivery system limits their therapeutic use as probiotics. In this study, we propose a novel nanofiber-based delivery system for the local administration of probiotics in the vagina. First, we tested the growth characteristics of three vaginal *Lactobacillus* species (*L. gasseri*, *L. crispatus*, and *L. jensenii*) in the presence of different polymers and disaccharides. We concluded that alginate and sucrose enhanced their growth and were therefore included in electrospinning formulations along with PEO as the carrier polymer. Sucrose preserved the viability of *Lactobacillus* species in the polymer solutions. It also improved the viability of *L. crispatus* and *L. jensenii* during electrospinning and of *L. gasseri* and *L. jensenii* during storage in nanofibers. The protective effect of sucrose can be attributed to its amorphization and interaction with the bacterial membrane. However, sucrose decreased the survival of *L. gasseri* immediately after electrospinning and of *L. crispatus* during storage. Nevertheless, all species survived the electrospinning in all formulations and retained viability for 56 days when encapsulated in nanofibers. Viability after 56 days differed among species, with *L. gasseri* showing the highest viability, followed by *L. jensenii* and *L. crispatus*, whereby the viability was also dependent on the excipients used for the bacterial encapsulation. In the present study, we determined that only an appropriate composition of the carrier system can result in the required viability of individual probiotics and that the composition must be tailored for each individual species. We showed that nanofibers are a suitable delivery system for vaginal probiotics and can be used for the development of a novel medicine for re-establishing the disturbed vaginal microbiota.

## Figures and Tables

**Table 1:** Species specific primers for colony PCR.

Primer Name	Primer Sequence (5′–3′)	Specificity	GenBank Target
*Lgas*-F	TCGTCGCGGTATTGAAACTG	*L. gasseri*	EF571590.1
*Lgas*-R	AAGGGTTGTCTAAGTCGGCT	*L. gasseri*	EF571590.1
*Lcri*-F	GCAGGCGATCGGATTCAAAT	*L. crispatus*	KF316678.1
*Lcri*-R	GGCCGTTGAAGTTTCTGGTT	*L. crispatus*	KF316678.1
*Ljen*-F	GGTCATGGTCTTGGTCTTGG	*L. jensenii*	CP018809.1
*Ljen*-R	GCAAATCATTGTGGTCAACG	*L. jensenii*	CP018809.1

**Table 2:** The theoretical and experimental enthalpy and moisture content of PEO, alginate (ALG), and sucrose (SUC), their physical mixture, nanofiber mats with and without bacteria, and lyophilized bacteria.

Sample	PEO Melting			Sucrose Melting			Moisture Content %
	Theoretical Enthalpy (J/g)	Enthalpy (J/g)	Peak Temperature (°C)	Theoretical Enthalpy (J/g)	Enthalpy (J/g)	Peak Temperature (°C)	
PEO	−186.8	−186.8	70.8	-	-	-	0
PEO nanofibers	−186.8	−109.4	66.4	-	-	-	0
ALG	-	-	-	-	-	-	12.3
PEO/ALG physical mixture (80/20)	−149.5	−152.81	70.6	-	-	-	-
PEO/ALG nanofibers (80/20)	−149.5	−81.1	60.4	-	-	-	2.4
SUC	-	-	-	−178.3	−178.3	191.9	0
PEO/ALG/SUC physical mixture (40/10/50)	−74.7	−66.6	70.5	−89.1	−92.5	193.1	-
PEO/ALG/SUC nanofibers (40/10/50)	−74.7	−40.9	56.7	−89.1	−33.4	187.3	1.2
*L. gasseri*	-	-	-	-	-	-	5.1
*L. gasseri* PEO nanofibers	−128.6	−76.8	73.9	-	-	-	2.8
*L. gasseri* PEO/ALG nanofibers	−91.2	−60.3	63.8	-	-	-	4.1
*L. gasseri* PEO/ALG/SUC nanofibers	−57.7	−30.9	63.3	−68.8	−7.2	173.4	3.5
*L. crispatus*	-	-	-	-	-	-	5.0
*L. crispatus* PEO nanofibers	−100.8	−84.8	70.9	-	-	-	2.6
*L. crispatus* PEO/ALG nanofibers	−79.2	−60.9	64.1	-	-	-	3.4
*L. crispatus* PEO/ALG/SUC nanofibers	−48.4	−27.9	63.2	−57.8	−6.4	167.9	3.5
*L. jensenii*	-	-	-	-	-	-	5.8
*L. jensenii* PEO nanofibers	−119.3	−83.8	71.9	-	-	-	1.7
*L. jensenii* PEO/ALG nanofibers	−84.1	−41.5	63.3	-	-	-	3.3
*L. jensenii* PEO/ALG/SUC nanofibers	−55.2	−27.1	59.7	−65.9	−17.8	183.4	2.4

## Data Availability

The datasets used and/or analyzed during the current study are available from the corresponding author on reasonable request.
